# Supplementary material for: Dual client binding sites in the ATP-independent chaperone SurA
Source: Nat Commun. 2024 Sep 14;15:8071. doi: 10.1038/s41467-024-52021-1 (PMC11401910; doi:10.1038/s41467-024-52021-1)
Supplement: Supplementary file 1 — Supplementary Information [file 41467_2024_52021_MOESM1_ESM.pdf]

Supplementary information

Dual client binding sites in the ATP-independent chaperone SurA

Bob Schiffrin[1*], Joel A. Crossley[1*], Martin Walko[1,2], Jonathan M. Machin[1], G. Nasir Khan[1], Iain W. Manfield[1], Andrew J. Wilson[2,3], David J. Brockwell[1], Tomas Fessl[4], Antonio N. Calabrese[1], Sheena E. Radford[1#], Anastasia Zhuravleva[1#]

[1]Astbury Centre for Structural Molecular Biology, School of Molecular and Cellular Biology, Faculty of Biological Sciences, University of Leeds, Leeds LS2 9JT

[2]Astbury Centre for Structural Molecular Biology, School of Chemistry, University of Leeds, Leeds LS2 9JT

[3]Current address: School of Chemistry, University of Birmingham, Edgbaston, Birmingham, B15 2TT, UK

[4]Faculty of Science, University of South Bohemia, Branišovská 1760, 370 05 České Budějovice, Czech Republic

*Equal contribution

#To whom correspondence should be addressed: s.e.radford@leeds.ac.uk and a.zhuravleva@leeds.ac.uk

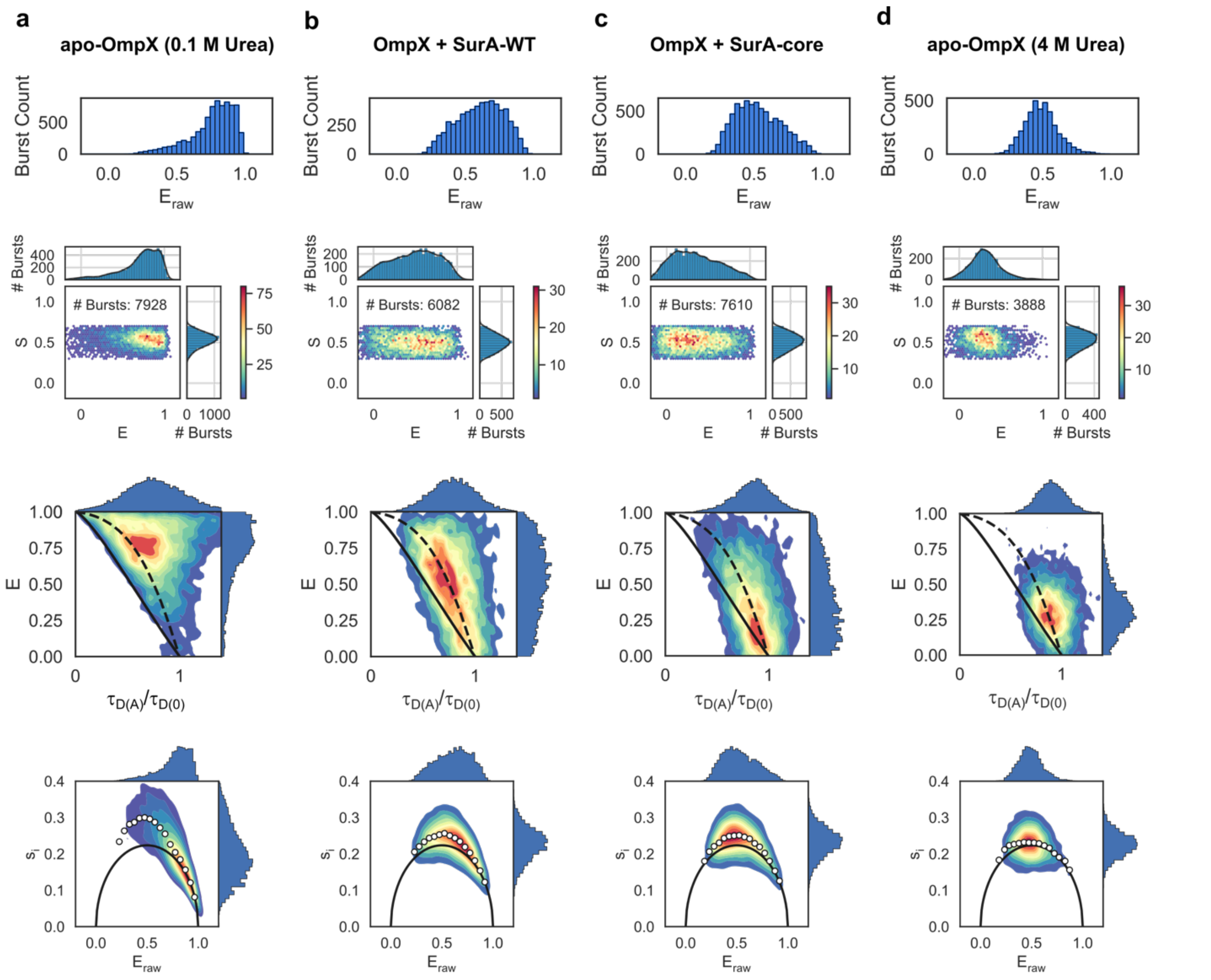

Supplementary Fig. 1. smFRET data for labelled OmpX in the presence or absence of SurA variants. Data are shown for (**a**) apo-OmpX (0.1 M urea), (**b**) OmpX + SurA-WT, (**c**) OmpX + SurA-core, and (**d**) apo-OmpX (4 M urea). The top row shows the uncorrected FRET efficiency histograms (E_{raw}) of filtered FRET bursts. The second row shows corrected FRET efficiency (E) vs stoichiometry (S) for the filtered FRET bursts. The third row shows plots of E versus normalised fluorescence donor lifetime ($\tau_{D(A)}/\tau_{D(0)}$) (Methods). The solid black line represents the correlation between these two values expected for a static (≤ ms timescale) FRET species. FRET populations which deviate to the right from this line exhibit dynamics. The dashed black line represents the expected correlation between E and $\tau_{D(A)}/\tau_{D(0)}$ for a rapidly interconverting Gaussian chain. The fourth row shows Burst Variance Analysis (BVA) where E_{raw} is plotted vs the standard deviation in the burst (s_i). The solid black line represents the expected standard deviation for a static (ms timescale) FRET population. The white circles show the mean s_i for bin widths of 0.05 of E_{raw}. Source data are provided as a Source Data file.

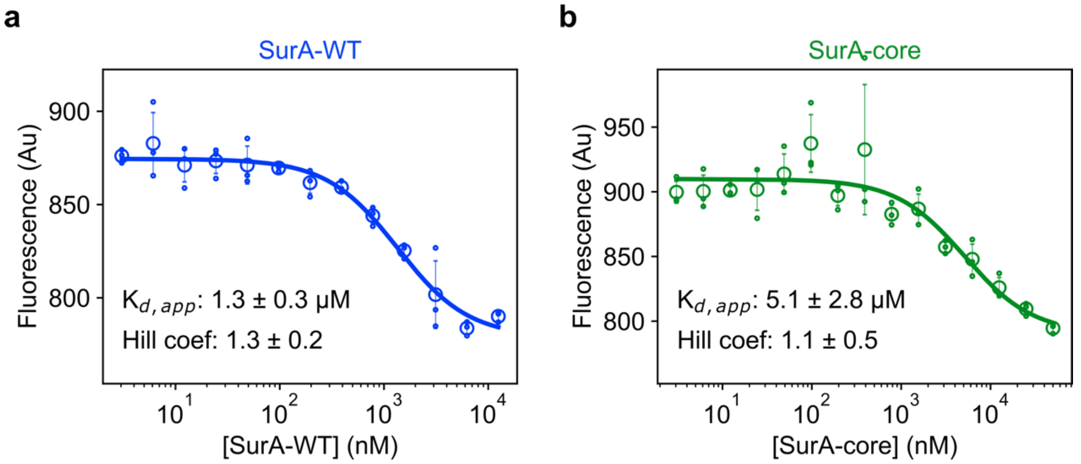

Supplementary Fig. 2. Microscale thermophoresis (MST) data for binding of OmpX to (**a**) SurA-WT, and (**b**) SurA-core. Samples contained 100 nM Alexa Fluor 488-labelled OmpX (Methods), SurA-WT (3 nM-50 μM), 0.8 M urea, 20 mM Tris-HCl, pH 8, at 25 °C. Three independent replicates were performed and averaged prior to fitting. The mean for each SurA-WT or SurA-core concentration is shown as open circles and the individual values for each replicate are shown as dots. The error bars represent the standard deviation between replicates. Data were fitted to a Hill binding equation (Methods). Source data are provided as a Source Data file.

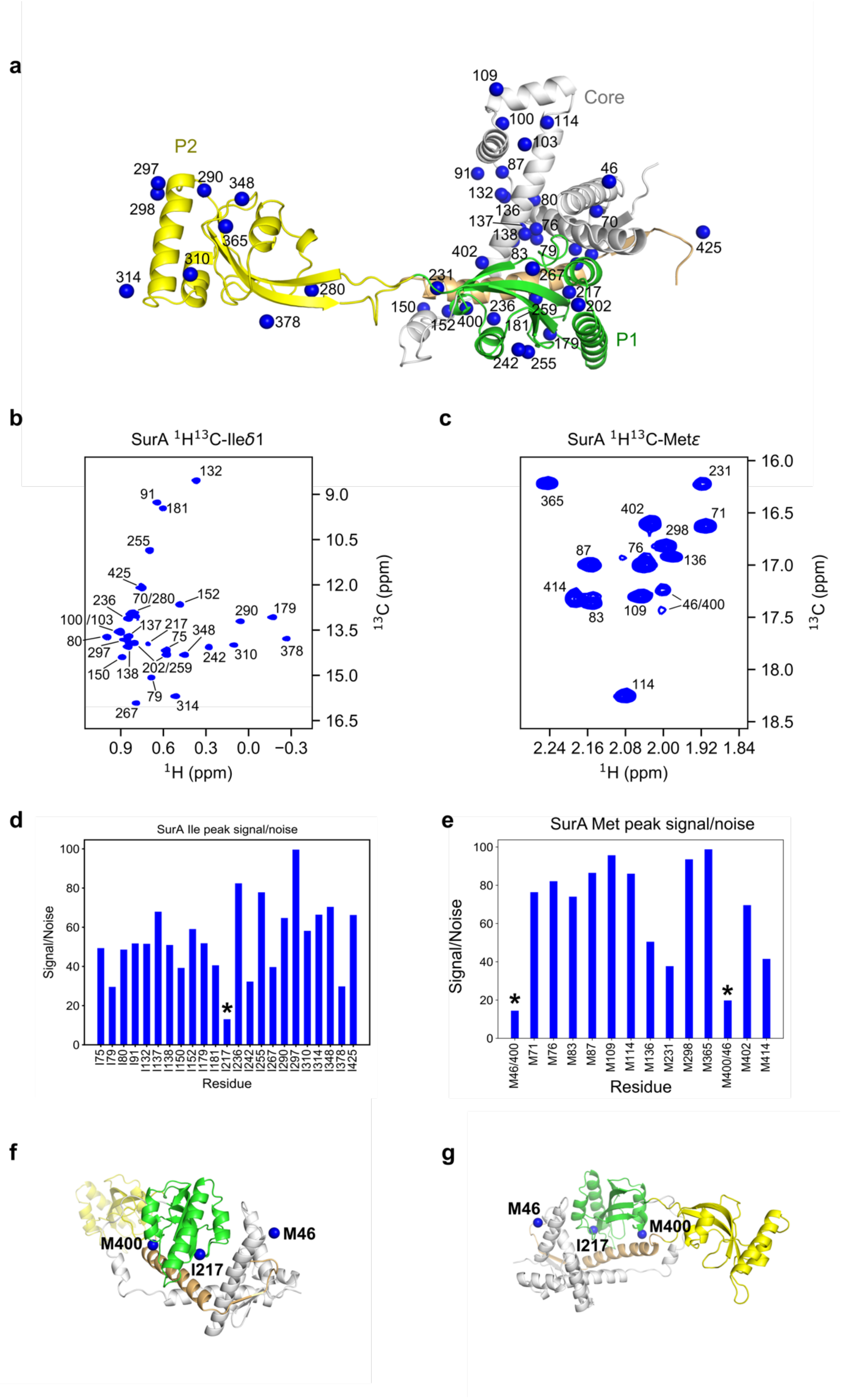

Supplementary Fig. 3. Peaks for Ile and Met residues located at, or near, the interface of the core and P1 domains in the crystal structure of SurA are the most broadened in the methyl-

TROSY NMR spectrum of SurA-WT. (**a**) Locations of Ile and Met residues mapped onto the crystal structure of SurA (PDB: 1M5Y[1]). NMR-active ^{13}C-labelled atoms for Ile (Cδ1) and Met (Cε) are highlighted as blue spheres. (**b**) Ile region, and (**c**) Met region of the NMR spectrum of apo-SurA (wild-type without client) with assignments. Two pairs of peaks are overlapped in the Ile spectrum (I70/I280) and (I100/I103). Peaks for I202/I259 and M46/M400 could not be unambiguously assigned (by mutating each residue individually (see Methods)). (**d**,**e**) Signal-to-noise ratios of peaks for (**d**) Ile and (**e**) Met in the NMR spectrum of apo-SurA. The most broadened peaks from residues in the core domain (M46, M400) and P1 domain (I217) are indicated by asterisks. Note that signal-to-noise ratios for peaks which are overlapped in the spectrum (corresponding to I70/I280 and I100/I103) could not be obtained and are omitted from (**d**). (**f,g**) Two views of SurA highlighting residues in the core domain (M46, M400) and P1 domain (I217) whose corresponding peaks in the methyl-TROSY NMR spectrum of SurA-WT are the most broadened. Note that M46 is not directly in contact with the P1 domain (green) in the P1-closed conformation in the crystal structure, and additional dynamic movements within the core domain may contribute to its reduced relative intensity. Samples contained 5 μM SurA, 5 mM, EDTA, 20 mM Tris-HCl, pH 8, at 25 ˚C. Source data are provided as a Source Data file.

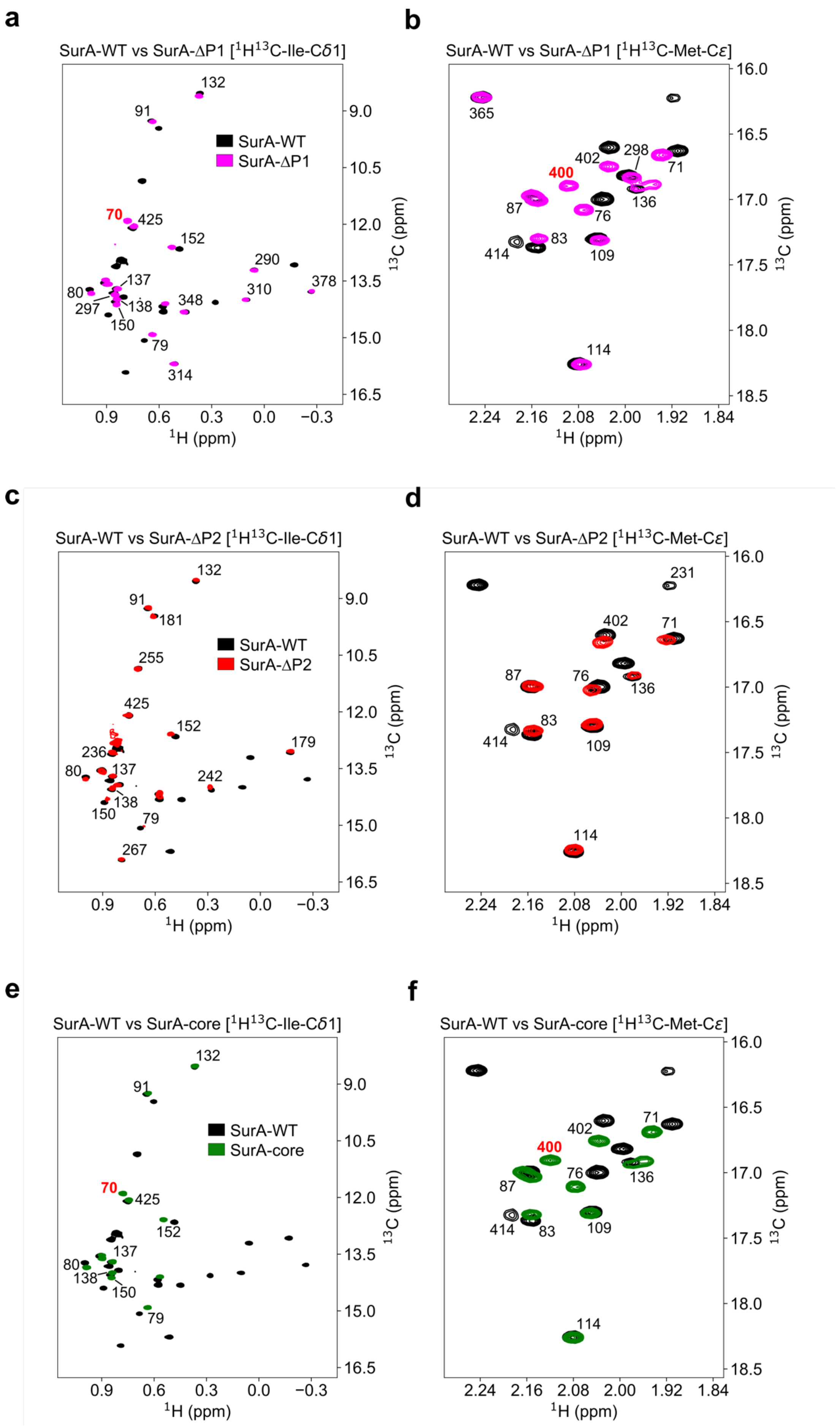

a
SurA-WT vs SurA-ΔP1 [¹H¹³C-Ile-Cδ1]
SurA-WT
SurA-ΔP1
132
91
70
425
152
290
310
378
137
80
297
138
348
150
79
314
0.9 0.6 0.3 0.0 −0.3
¹H (ppm)
9.0 10.5 12.0 13.5 15.0 16.5
¹³C (ppm)
b
SurA-WT vs SurA-ΔP1 [¹H¹³C-Met-Cε]
365
402
298
400
71
87
76
136
83
414
109
114
2.24 2.16 2.08 2.00 1.92 1.84
¹H (ppm)
16.0 16.5 17.0 17.5 18.0 18.5
¹³C (ppm)
c
SurA-WT vs SurA-ΔP2 [¹H¹³C-Ile-Cδ1]
SurA-WT
SurA-ΔP2
132
91
181
255
425
152
179
236
80
137
242
138
150
79
267
0.9 0.6 0.3 0.0 −0.3
¹H (ppm)
9.0 10.5 12.0 13.5 15.0 16.5
¹³C (ppm)
d
SurA-WT vs SurA-ΔP2 [¹H¹³C-Met-Cε]
231
402
71
87
76
136
83
414
109
114
2.24 2.16 2.08 2.00 1.92 1.84
¹H (ppm)
16.0 16.5 17.0 17.5 18.0 18.5
¹³C (ppm)
e
SurA-WT vs SurA-core [¹H¹³C-Ile-Cδ1]
SurA-WT
SurA-core
132
91
70
425
152
137
80
138
150
79
0.9 0.6 0.3 0.0 −0.3
¹H (ppm)
9.0 10.5 12.0 13.5 15.0 16.5
¹³C (ppm)
f
SurA-WT vs SurA-core [¹H¹³C-Met-Cε]
402
71
400
76
87
136
414
83
109
114
2.24 2.16 2.08 2.00 1.92 1.84
¹H (ppm)
16.0 16.5 17.0 17.5 18.0 18.5
¹³C (ppm)

Supplementary Fig. 4. SurA domain deletion variants (**Fig. 1d**) suggest extensive interactions between SurA P1 domain and the core domain in SurA-WT. Methyl-TROSY NMR spectra of SurA-WT compared with (**a,b**) SurA-ΔP1, (**c,d**) SurA-ΔP2, and (**e,f**) SurA-core. Ile and Met regions are shown in (**a,c,e**) and (**b,d,f**), respectively. Spectra for SurA-WT, SurA-ΔP1, SurA-ΔP2, and SurA-core are coloured in black, magenta, red, and green, respectively. Two peaks for residues in the core domain (I70 and M400), which are highly broadened in the spectrum of SurA-WT, appear in the spectra for SurA variants lacking P1, and are highlighted in red font. Note the broadening of the peak for M414 which lies in SurA core in the spectra of all domain deletion variants. Samples contained 5 μM SurA or SurA variant, 5 mM, EDTA, 20 mM Tris-HCl, pH 8, at 25 ˚C.

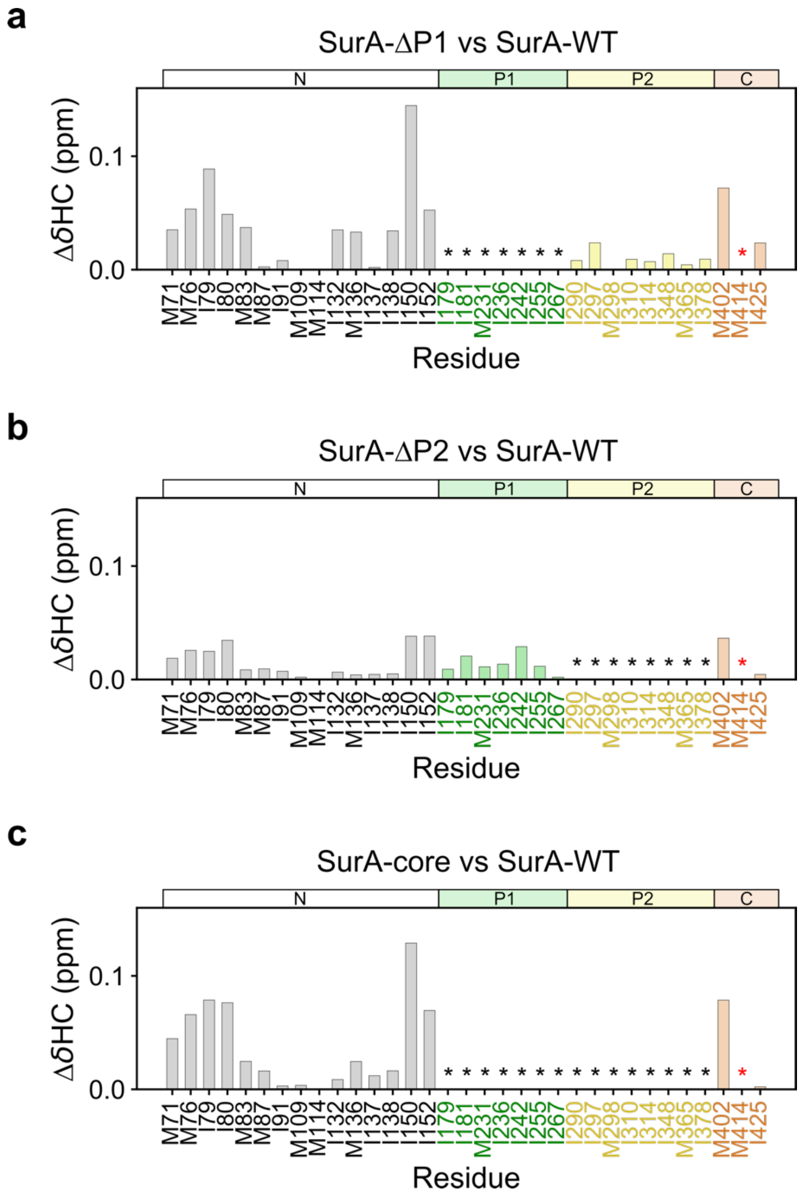

Supplementary Fig. 5. Chemical shift perturbations (CSPs) in the SurA core domain are greater in domain deletion variants lacking P1 (SurA-ΔP1 and SurA-core) compared with the P2 deletion construct (SurA-ΔP2). CSP plots are shown for (**a**) SurA-ΔP1 vs SurA-WT, (**b**) SurA-ΔP2 vs SurA-WT, and (**c**) SurA-core vs SurA-WT. Residue M414 in the core domain (red asterisk) is broadened in all variants. Residues which are not present in domain deletion constructs are indicated by black asterisks. Source data are provided as a Source Data file.

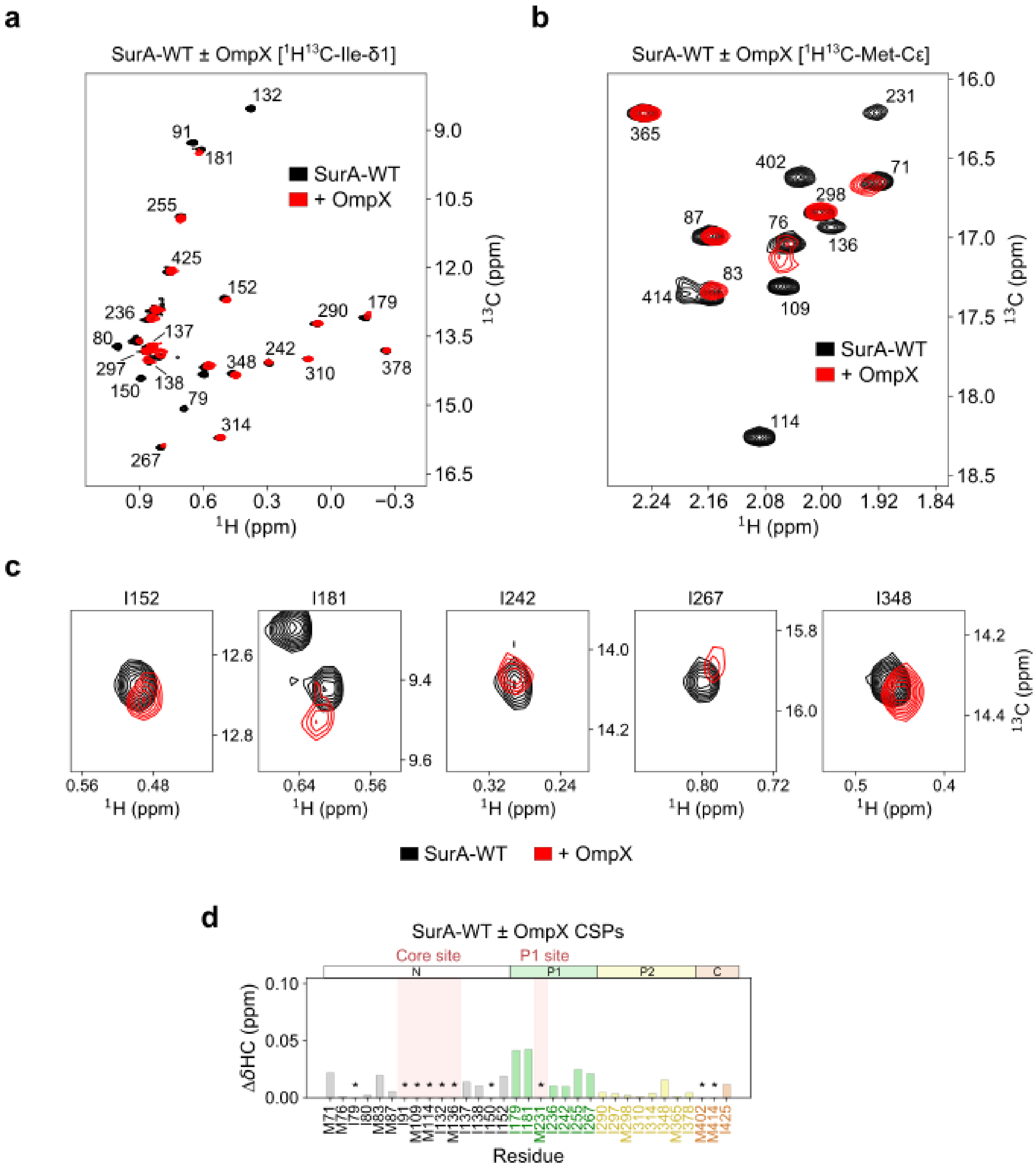

Supplementary Fig. 6. Interaction of SurA-WT with unfolded OmpX by methyl-TROSY NMR. (**a**,**b**) NMR spectra of SurA-WT alone (black) and with the addition of equimolar OmpX (red). Regions of the spectra containing (**a**) Ile peaks or (**b**) Met peaks are shown. (**c**) Zoomed in regions of spectra from (**a**) showing example CSPs in the P1 (I152, I181, I242, I267) and P2 (I348) domains. (**d**) CSPs for SurA-WT peaks in the presence of OmpX are shown, with residues broadened to below the noise indicated by an asterisk. Pink blocks indicate residues located in core and P1 binding hotspots, and are similarly highlighted in subsequent figures. Note that for apo-SurA-WT the broadened peaks for M400/M46 are not present in these

experiments in the presence of 0.8 M urea, and with shorter acquisitions compared to non-OMP containing experiments (see Methods). Samples contained 5 μM SurA, ± 5 μM OmpX, 0.8 M urea, 5 mM, EDTA, 20 mM Tris-HCl, pH 8, at 25 ˚C. Source data are provided as a Source Data file.

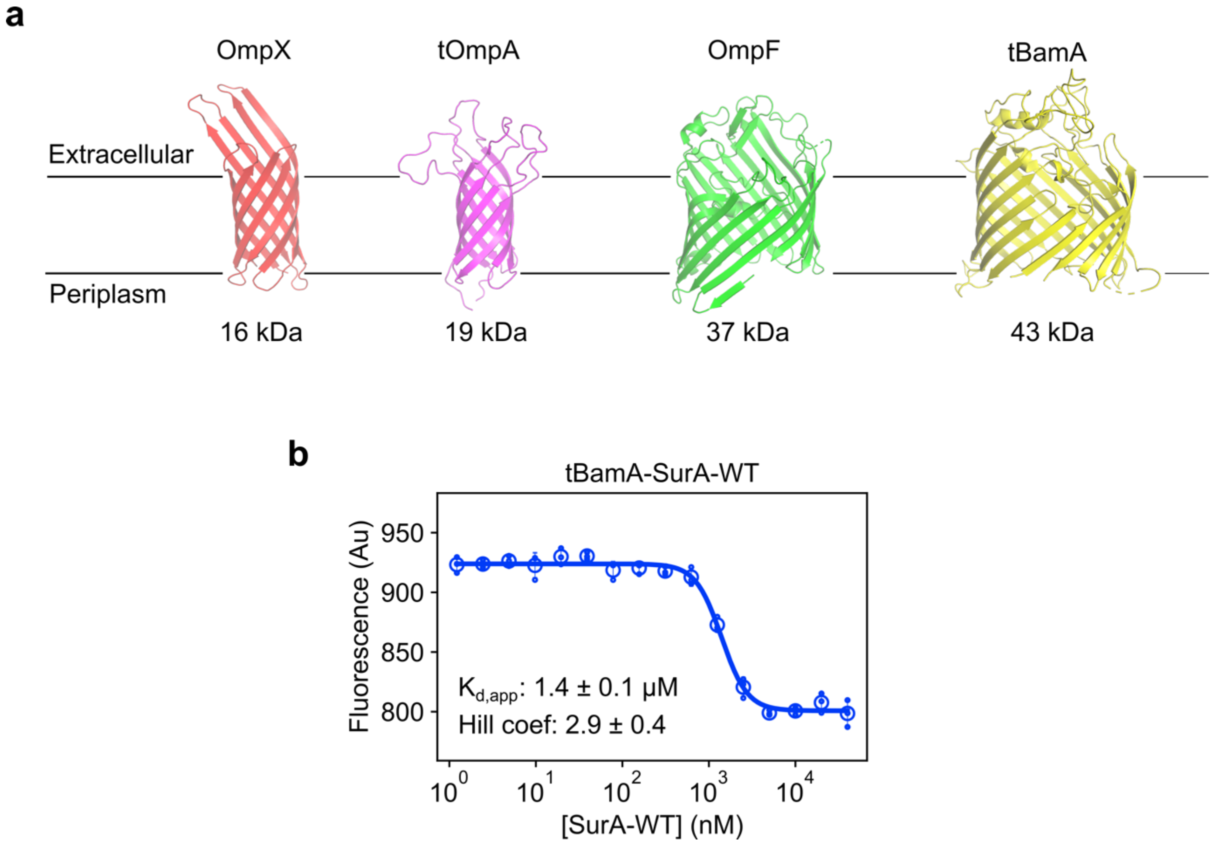

Supplementary Fig. 7. OMPs of different size and sequence used in this study. (**a**) Structures (from left to right) of natively folded OmpX (PDB: 1QJ8[2]), tOmpA (PDB: 1G90[3]) OmpF (PDB: 2ZFG[4]) and tBamA (PDB: 4N75[5]). All OMPs are shown to scale. Note that OmpF is trimeric *in vivo*, a single subunit is shown here for direct size comparison. (**b**) Microscale thermophoresis (MST) data for binding of tBamA to SurA-WT. Samples contained 100 nM Alexa Fluor 488-labelled tBamA (Methods), SurA-WT (1.2 nM-40 μM), 20 mM Tris-HCl, pH 8, at 25 °C. Three independent replicates were performed and averaged prior to fitting. The mean for each SurA-WT concentration is shown as open circles and the individual values for each replicate are shown as dots. The error bars represent the standard deviation between replicates. Data were fitted to a Hill binding equation (see Methods). Source data are provided as a Source Data file.

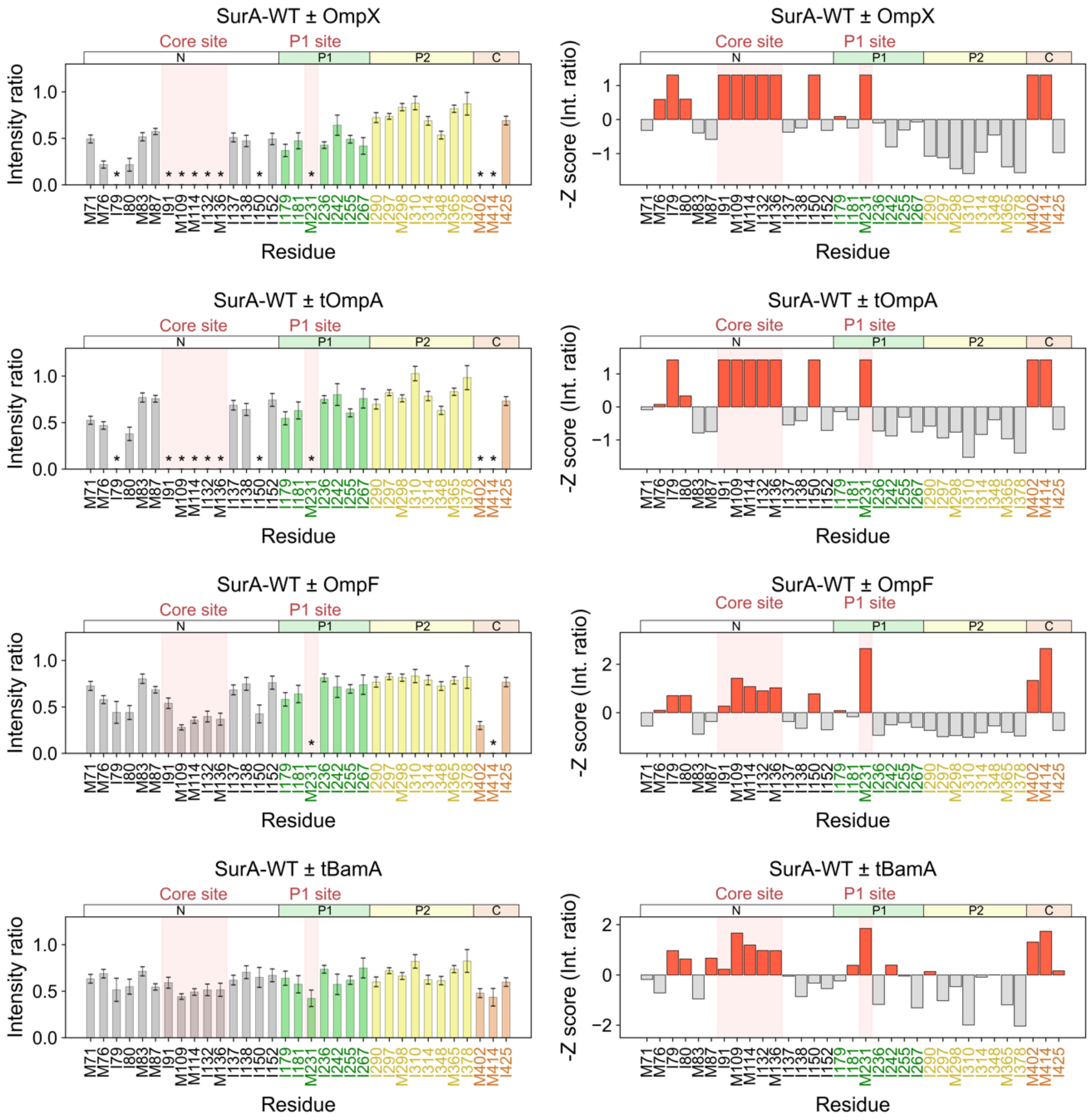

Supplementary Fig. 8. OMPs of different size and sequence interact with similar locations on the surface of SurA. Changes in intensity ratios and the Z-scores of the intensity ratio changes are shown for SurA binding to unfolded OmpX, tOmpA, OmpF and tBamA. Each substrate was unfolded in 8M urea and diluted into SurA-WT to form the bound complex. Note that the absolute reductions in intensities are smaller for the larger OMPs, presumably because of differences in dynamics of the longer chains. Nonetheless, the locations affected are similar. Samples contained 5 μM SurA, ± 5 μM OMP, 0.8 M urea, 5 mM, EDTA, 20 mM Tris-HCl, pH 8, at 25 ˚C. Int: Intensity. Signal to noise ratios were used for the calculation of errors in peak intensities in intensity ratio panels (left) (Methods). Peaks which are broadened below the noise in the intensity ratio plots are indicated by an asterisk Source data are provided as a Source Data file.

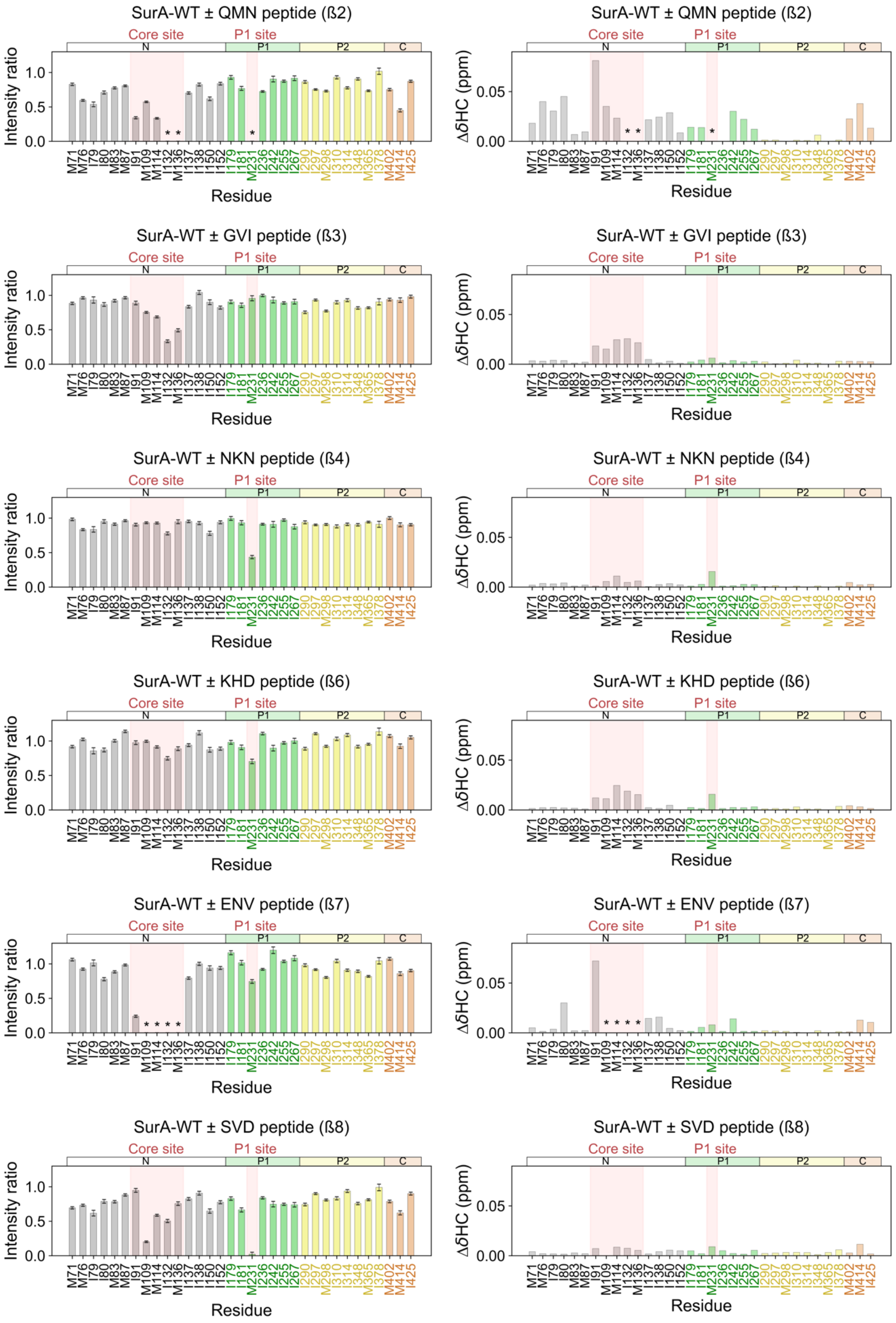
SurA-WT ± QMN peptide (ß2)
SurA-WT ± GVI peptide (ß3)
SurA-WT ± NKN peptide (ß4)
SurA-WT ± KHD peptide (ß6)
SurA-WT ± ENV peptide (ß7)
SurA-WT ± SVD peptide (ß8)
Core site
P1 site
N
P1
P2
C
Intensity ratio
ΔδHC (ppm)
Residue
M71 M76 I79 I80 M83 M87 I91 M109 M114 I132 M136 I137 I138 I150 I152 I179 I181 M231 I236 I242 I255 I267 I290 I297 M298 I310 I314 I348 M365 I378 M402 M414 I425

Supplementary Fig. 9. Intensity ratio and CSP data for SurA-WT binding to OmpX-derived peptides. Data are shown for peptide concentrations of 200 μM except for the terminal β-strand peptide (SVD (β8)), which was not soluble in NMR buffer at this concentration and therefore contained 50 μM peptide. Each 15-residue peptide sequence corresponds to a different β-strand in the OmpX native state (**Fig. 3a,b**), with the strand number indicated in brackets. Samples contained 5 μM SurA, ± 50 or 200 μM OmpX-derived peptide (as detailed above), 0.8 M urea, 5 mM, EDTA, 20 mM Tris-HCl, pH 8, at 25 ˚C. Signal to noise ratios were used for the calculation of errors in peak intensities in intensity ratio panels (left) (Methods). Peaks which are broadened below the noise in the intensity ratio plots are indicated by an asterisk Source data are provided as a Source Data file.

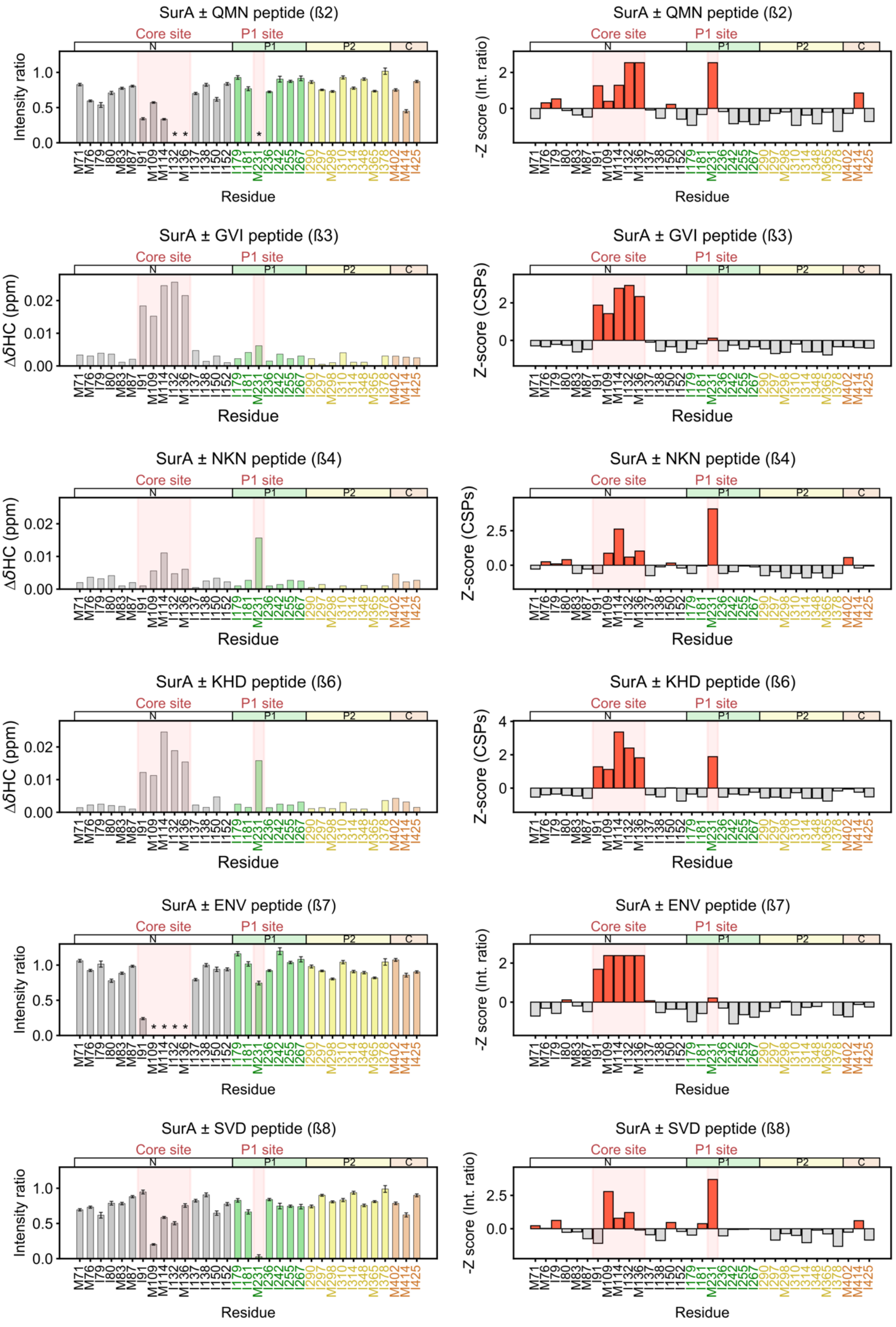

Supplementary Fig. 10. OmpX-derived peptides containing Ar-X-Ar or Ar-Ar motifs interact with two binding hotspots in the core and P1 domains of SurA-WT. Intensity ratio data are

shown for the QMN (β2), ENV (β7), and SVD (β8) peptides. For the GVI (β3), NKN (β4) and KHD (β6) peptides, the changes upon binding are mostly reflected in CSPs. Intensity ratio and CSP data for all peptides are shown in **Supplementary Fig. 9**. Peaks which are broadened below the noise in the intensity ratio plots are indicated by an asterisk. For these peaks, the noise value for the bound spectra were used in the calculation of Z-scores. Data are shown for peptide concentrations of 200 μM, except for SVD (β8), which contained 50 μM due to its solubility limits. Each 15-residue peptide sequence corresponds to a different β-strand in the OmpX native state (**Fig. 3a,b**), with the strand number indicated in brackets. Samples contained 5 μM SurA, ± 50 or 200 μM OmpX-derived peptide (as detailed above), 0.8 M urea, 5 mM, EDTA, 20 mM Tris-HCl, pH 8, at 25 ˚C. Int: Intensity. Signal to noise ratios were used for the calculation of errors in peak intensities in intensity ratio panels (left) (Methods). Source data are provided as a Source Data file.

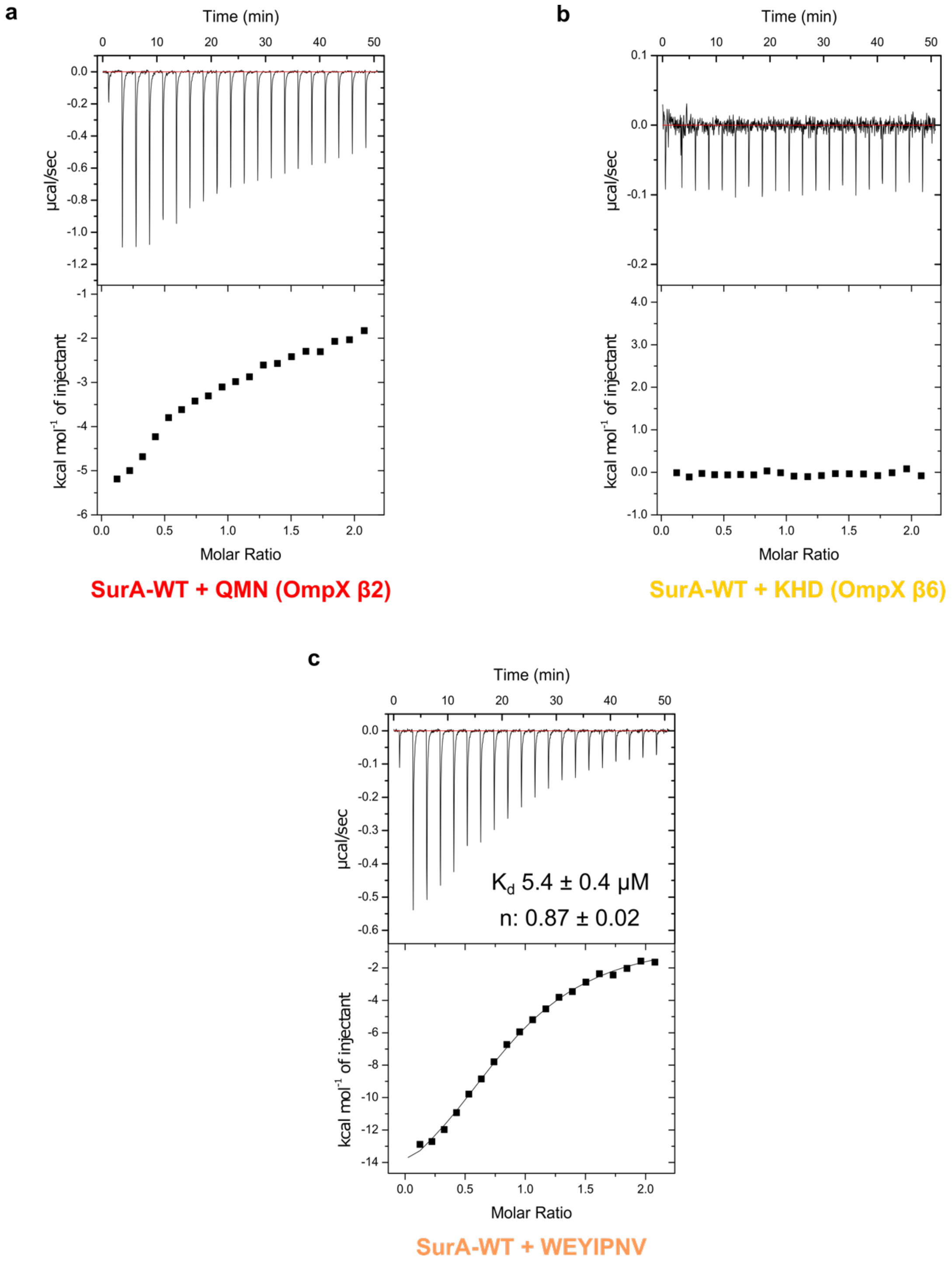

Supplementary Fig. 11. OmpX-derived peptides bind SurA-WT with weak affinity. ITC measurements for two example peptides (**a**) QMN (β2) and (**b**) KHD (β6). A full binding curve was not able to be obtained for any OmpX-derived peptide indicating weak affinities (>100 µM). (**c**) For comparison, ITC data for binding of the WEYIPNV peptide to SurA-WT is shown, yielding an affinity consistent with literature values[6]. In (**a**) and (**b**) concentrations of 100 µM SurA in the cell and 1 mM OmpX-derived peptides in the syringe were used. For (**c**)

concentrations of 20 μM SurA in the cell and 200 μM WEYIPNV peptide in the syringe were used. All samples contained 20 mM Tris-HCl, pH 8, and experiments were performed at 25 ˚C. Source data are provided as a Source Data file.

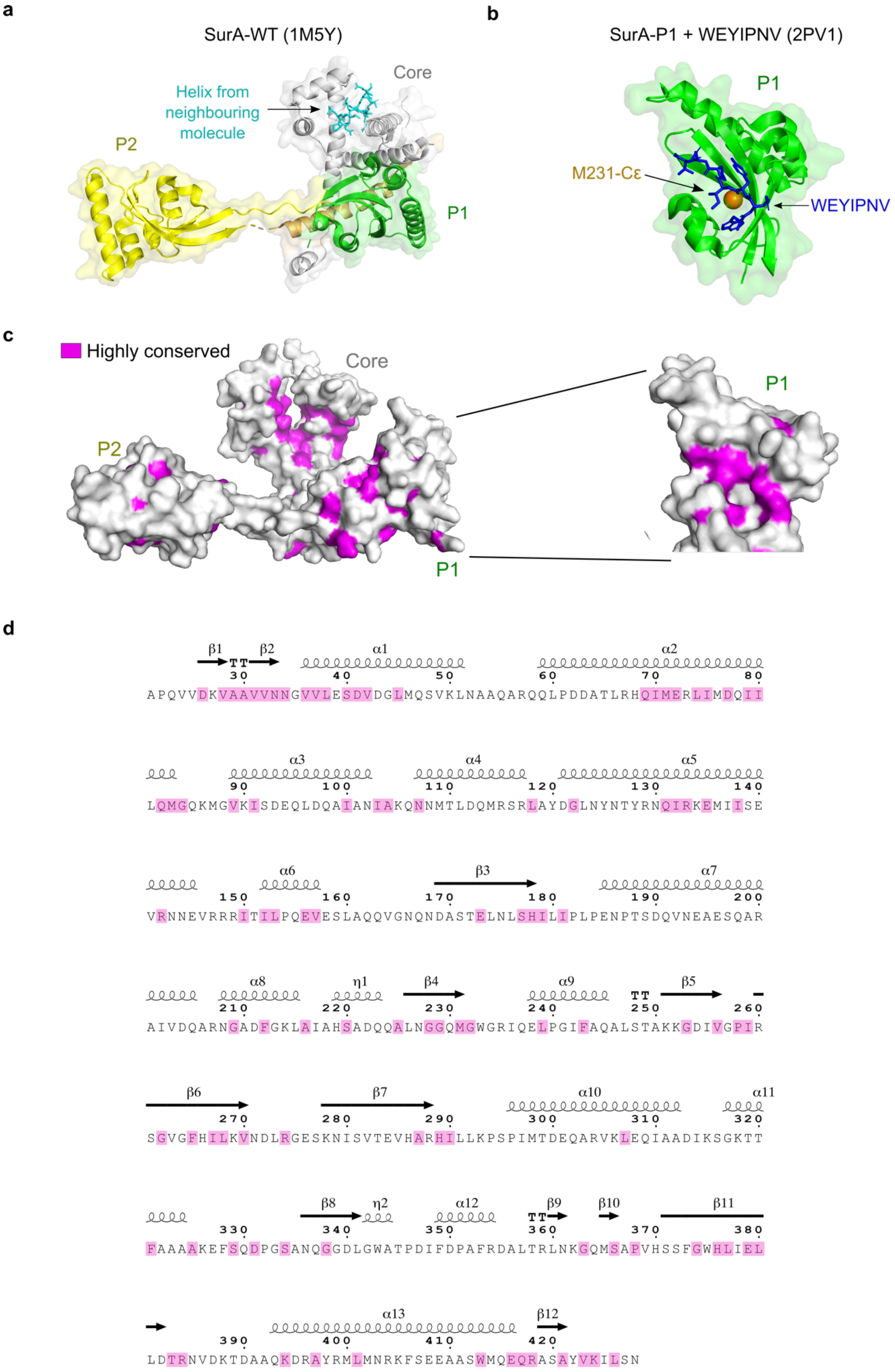
a
SurA-WT (1M5Y)
Helix from neighbouring molecule
Core
P2
P1
b
SurA-P1 + WEYIPNV (2PV1)
P1
M231-Cε
WEYIPNV
c
Highly conserved
Core
P2
P1
P1
d
β1 β2 α1 α2
30 40 50 60 70 80
APQVVDKVAAVVNNGVVLESDVDGLMQSVKLNAAQARQQLPDDATLRHQIMERLIMDQII
α3 α4 α5
90 100 110 120 130 140
LQMGQKMGVKISDEQLDQAIANIAKQNNMTLDQMRSRLAYDGLNYNTYRNQIRKEMIISE
α6 β3 α7
150 160 170 180 190 200
VRNNEVRRRITILPQEVESLAQQVGNQNDASTELNLSHILIPLPENPTSDQVNEAESQAR
α8 η1 β4 α9 β5
210 220 230 240 250 260
AIVDQARNGADFGKLAIAHSADQQALNGGQMGWGRIQELPGIFAQALSTAKKGDIVGPIR
β6 β7 α10 α11
270 280 290 300 310 320
SGVGFHILKVNDLRGESKNISVTEVHARHILLKPSPIMTDEQARVKLEQIAADIKSGKTT
β8 η2 α12 β9 β10 β11
330 340 350 360 370 380
FAAAAKEFSQDPGSANQGGDLGWATPDIFDPAFRDALTRLNKGQMSAPVHSSFGWHLIEL
α13 β12
390 400 410 420
LDTRNVDKTDAAQKDRAYRMLMNRKFSEEAASWMQEQRASAYVKILSN

Supplementary Fig. 12. The two binding hotspots in SurA-WT contain highly conserved residues. (**a**) Crystal structure of SurA-WT (PDB: 1M5Y[1]) highlighting a short polypeptide sequence from a neighbouring molecule in the crystal structure (cyan) located in the core domain binding crevice. (**b**) Crystal structure of the WEYIPNV peptide (blue) bound to the SurA P1 domain (green) (PDB: 2PV1[7]). The location of the Cε atom of M231 is highlighted as an orange sphere. (**c**) Analysis of SurA three domain homologues (Methods) indicates high conservation for residues within the two binding sites identified here using NMR. Highly conserved residues (those with a ConSurf score of 7/9 or higher) are highlighted in magenta. The P1 domain alone is shown on the right, rotated to visualise residues in the binding pocket. Highly conserved residues in P2 are located in the catalytic site. (**d**) Primary sequence of SurA-WT with highly conserved residues highlighted in magenta as in (**c**).

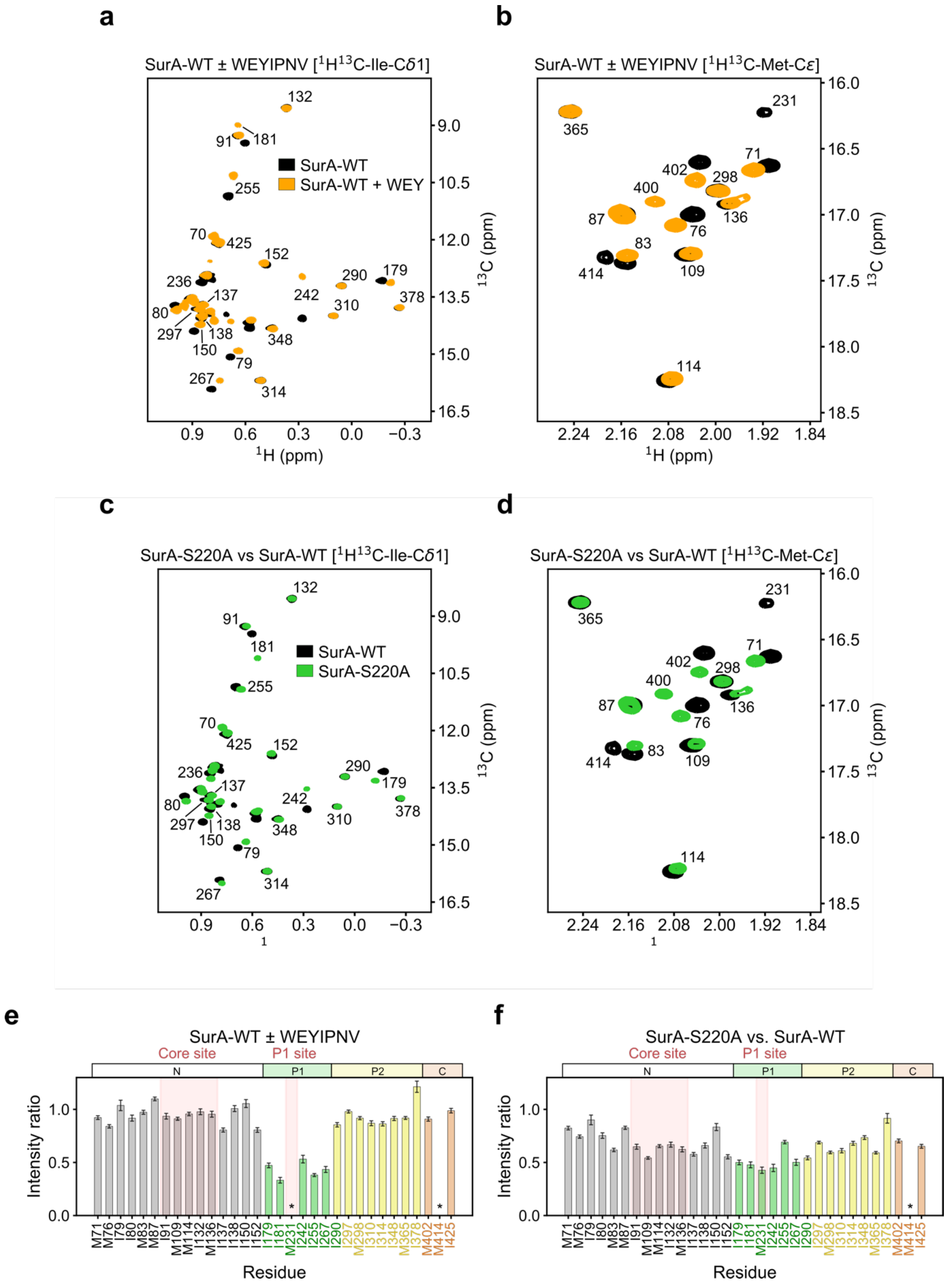

(Figure continued on next page)

g

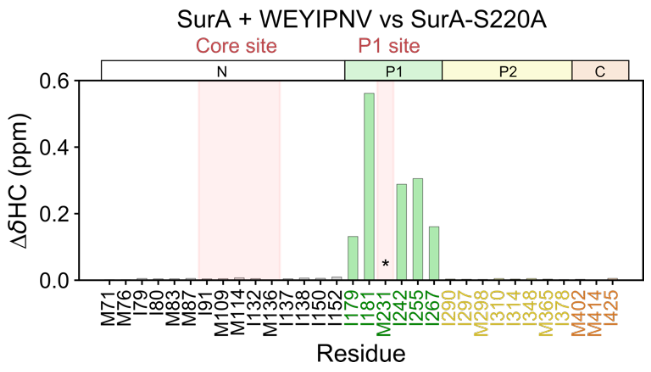

Supplementary Fig. 13. Binding of the WEYIPNV peptide to SurA-WT and the SurA-S220A variant cause similar conformational changes. Methyl-TROSY NMR spectra of (**a**,**b**) SurA-WT bound to WEYIPNV (orange) compared with SurA-WT alone (black) and (**c**,**d**) SurA-S220A (green) compared with SurA-WT alone (black). Ile and Met regions are shown in (**a**,**c**) and (**b**,**d**), respectively. (**e**,**f**) Intensity ratio changes between WEYIPNV-SurA-WT and SurA-WT, and SurA-S220A and SurA-WT, respectively. (**g**) Chemical shift differences between spectra of SurA-WT bound to WEYIPNV and SurA-WT vs. SurA-S220A, indicating similar chemical environments for core domain peaks in both cases. Resonances that have different chemical shifts in P1 are hence indicative of direct peptide binding. The peak corresponding to I236 in the spectrum of SurA-WT could not be confidently assigned for WEYIPNV-SurA-WT or SurA-S220A and so was excluded. Peaks which are broadened below the noise are indicated by an asterisk. Samples contained 5 μM SurA-WT ± 50 μM WEYIPNV or 5 μM SurA-S220A, 5 mM EDTA, 20 mM Tris-HCl, pH 8, at 25 ˚C. Signal to noise ratios were used for the calculation of errors in peak intensities in (e) and (f) (Methods). Source data are provided as a Source Data file.

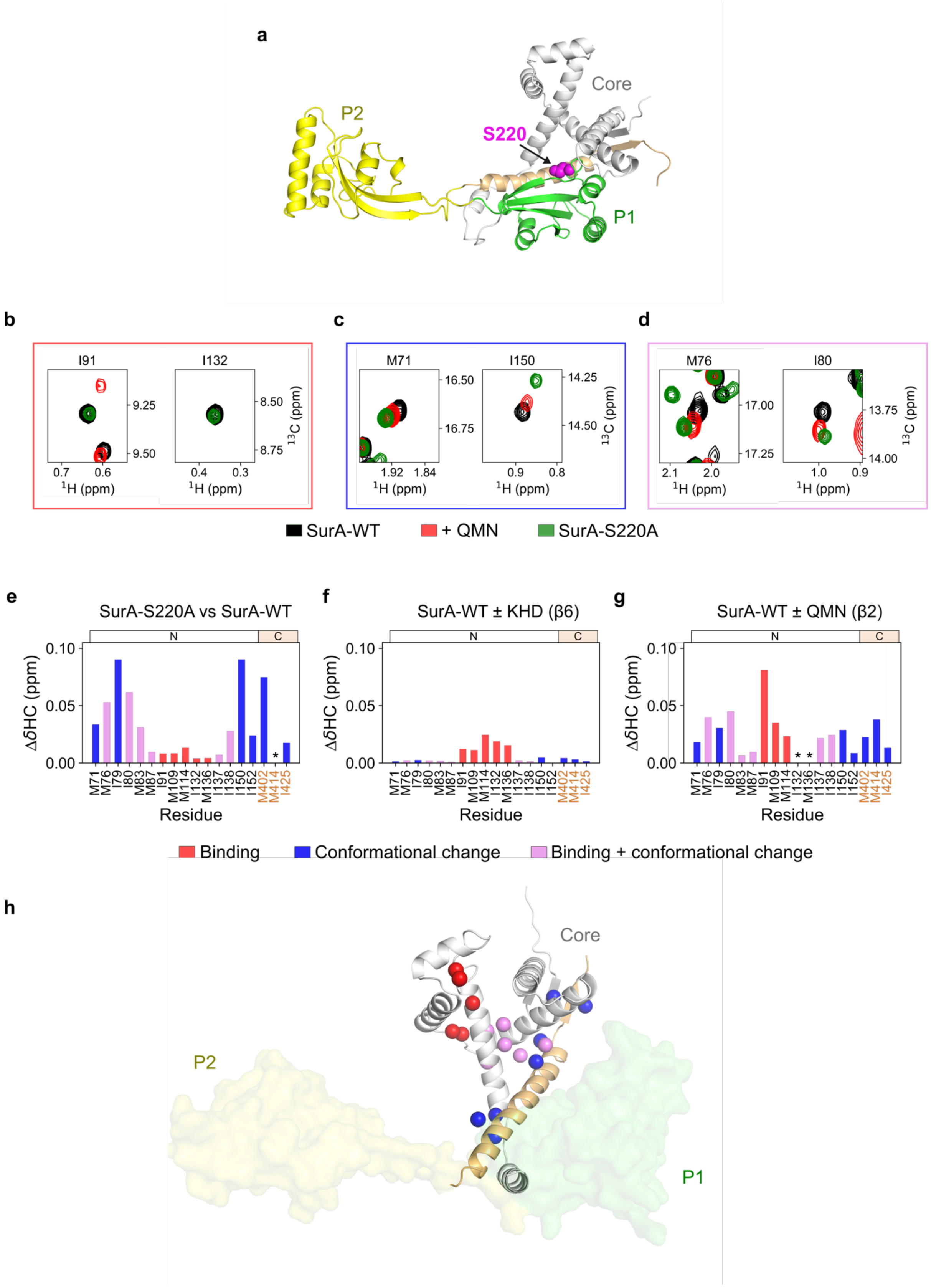

Supplementary Fig. 14. Binding of a peptide sequence derived from OmpX promotes a conformational rearrangement between the core and P1 domains to activate the chaperone.

(**a**) Residue S220 is located close to the core-P1 interface in the SurA core-P1 closed conformation (PDB: 1M5Y[1]). (**b-d**) Example zoomed in spectra comparing peaks in the spectra of SurA-WT alone, SurA-WT with the QMN (β2) peptide (200 µM), and SurA-S220A alone, which we classify as reporting mainly on (**b**) binding, (**c**) conformational changes, or (**d**) binding and conformational changes. Peaks were classified as reporting on conformational changes if the direction of the CSP upon binding of QMN to SurA-WT is identical to that between SurA-WT and SurA-S220A. Peaks were classified as reporting on binding and conformational changes if the CSP direction is similar, but not identical. (**e-g**) Zoom in on CSP plots showing core domain residues only for (**e**) SurA-S220A, (**f**) SurA-WT binding to the KHD (β6) peptide (200 µM), and (**g**) SurA-WT binding to the QMN (β2) peptide (200 µM). Bars are coloured by whether each peak reports largely on binding (red), conformational changes (blue), or binding and changes (violet), as judged by their behaviour in the NMR spectra. (**h**) Model of SurA in a core-P1 open conformation, highlighting which NMR probes in the core domain report on binding and/or conformational changes, coloured as in (**f-h**). Note that peaks which only report on conformational change (coloured blue) are located away from the binding site, and are close to the core-P1 interface. Peaks which are broadened below the noise are indicated by an asterisk. Source data are provided as a Source Data file.

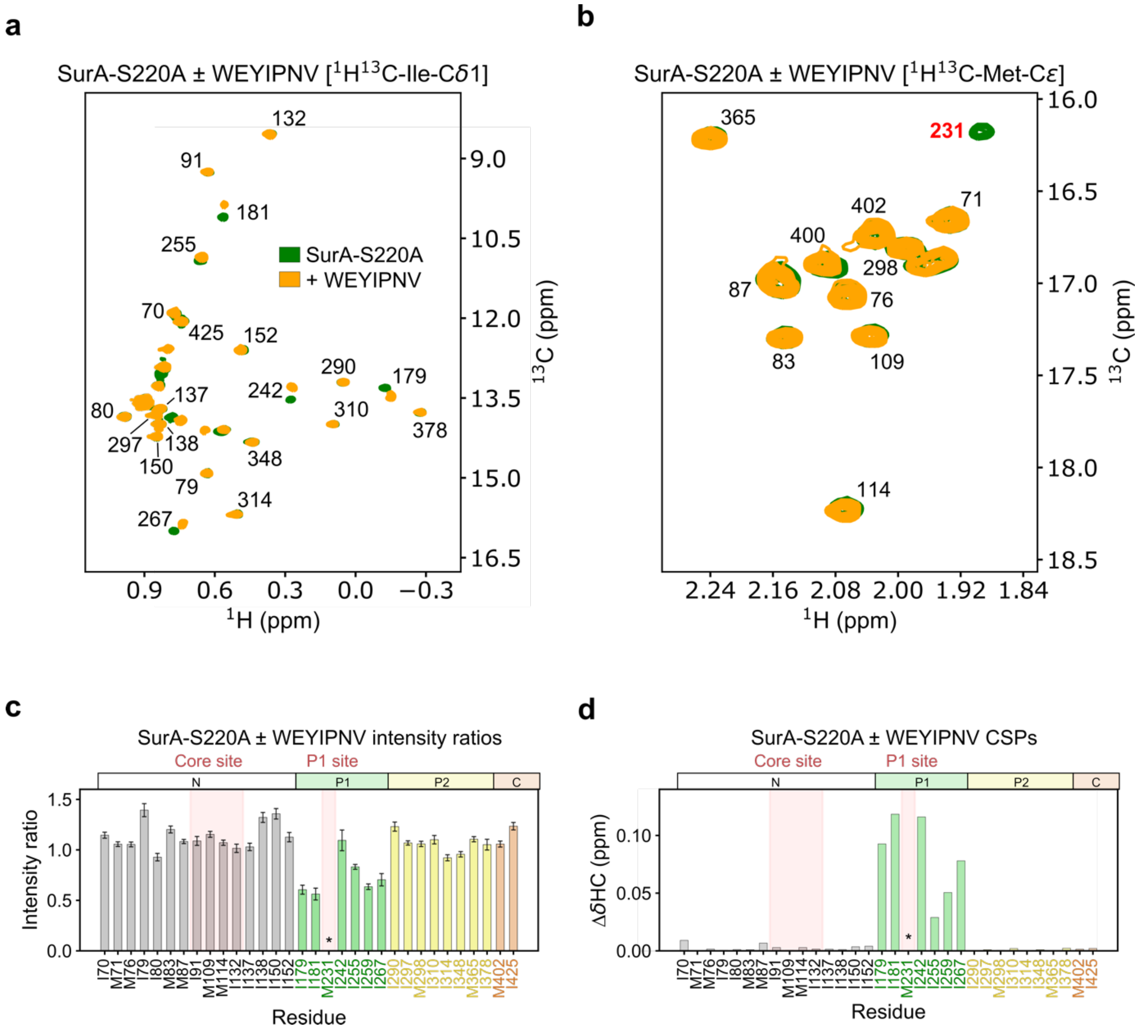

Supplementary Fig. 15. Loss of intensity of M231 peak in the methyl-TROSY NMR spectrum of SurA results from interaction with substrates. (**a,b**) Methyl-TROSY NMR spectra of SurA-S220A bound to WEYIPNV (orange) compared with SurA-S220A alone (green). Ile and Met regions are shown in (**a**) and (**b**), respectively. The peak for M231 which is broadened in the presence of WEYIPNV is highlighted in red in (**b**) and highlighted with an asterisk in (**c**) and (**d**). (**c**) Intensity ratios, and (**d**) CSPs between SurA-S220A alone and SurA-S220A bound to WEYIPNV. The peak corresponding to I236 in the spectrum of SurA-WT could not be confidently assigned for WEYIPNV-SurA-WT or SurA-S220A and so was excluded. The peak for M414 is broadened in the spectra of both WEYIPNV-SurA-WT and SurA-S220A so is not included in (**c**) and (**d**). Samples contained 5 μM SurA-S220A ± 50 μM WEYIPNV, 5 mM EDTA, 20 mM Tris-HCl, pH 8, at 25 ˚C. Signal to noise ratios were used for the calculation of errors in peak intensities in (c) (Methods). Source data are provided as a Source Data file.

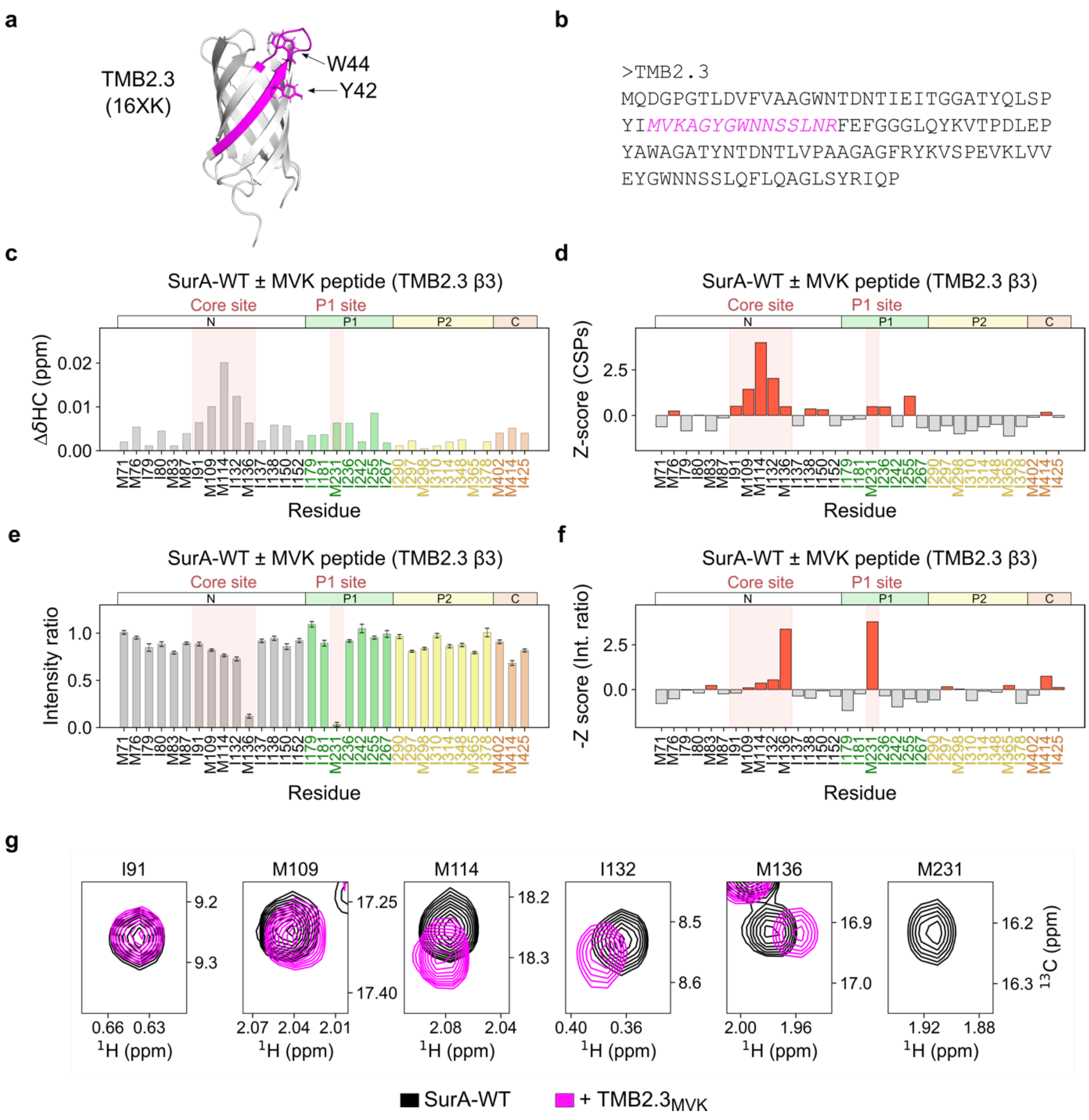

Supplementary Fig. 16. A peptide from the *de novo* designed transmembrane β-barrel TMB2.3[8] containing an Ar-X-Ar motif interacts with the identified OMP binding sites in the core and P1 domains of SurA. (**a**) Solution NMR structure of TMB2.3 (PDB: 16XK[8]). The 15-residue peptide beginning with the amino acids MVK (TMB2.3 β3) is highlighted in magenta on the structure. The aromatic residues of the Ar-X-Ar motif in this peptide (Y42 and W44) are indicated as sticks. (**b**) Primary sequence of TMB2.3 with the MVK peptide (TMB2.3 β3) highlighted in magenta. (**c**) CSPs and (**d**) Z-scores of the CSP data for the interaction between MVK (TMB2.3 β3) and SurA-WT. (**e**) Intensity ratios and (**f**) Z-scores of the intensity ratio data for the interaction of between MVK (TMB2.3 β3) and SurA-WT. (**g**) Zoomed in regions of NMR spectra for SurA alone (black), or SurA-WT in the presence of TMB2.3$_{MVK}$ (magenta)

highlighting regions of the spectra for residues indicative of interaction with the core (I91, M109, M114, I132, and M136), or P1 (M231) binding hotspots. Samples contained 5 μM SurA ± 200 μM TMB2.3$_{MVK}$, 5 mM EDTA, 20 mM Tris-HCl, pH 8, at 25 ˚C. Signal to noise ratios were used for the calculation of errors in peak intensities in (e) (Methods). Source data are provided as a Source Data file.

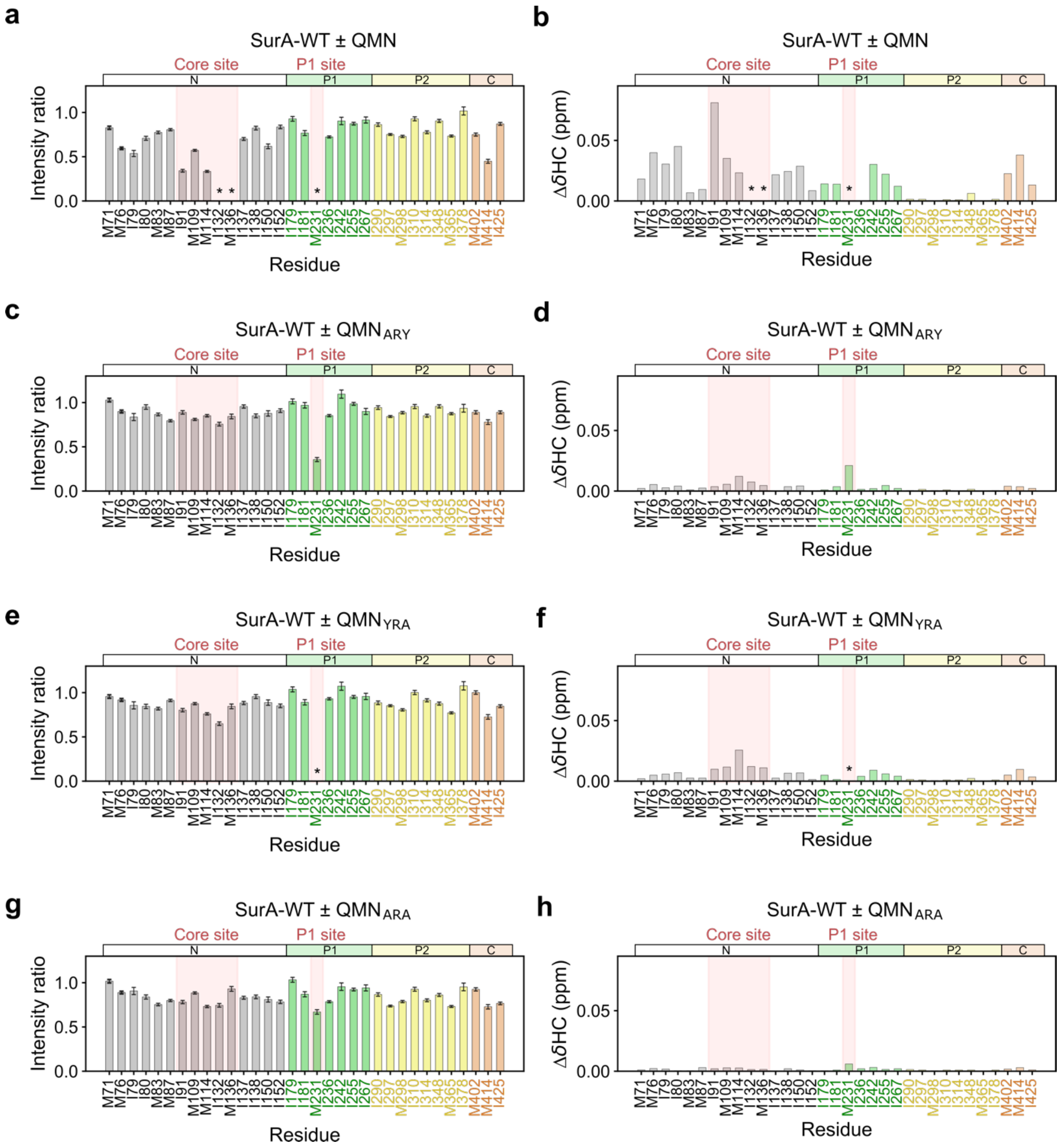

Supplementary Fig. 17. Mutation of the Ar-X-Ar motif abolishes binding of an OmpX-derived peptide to SurA-WT. Intensity ratio (**a,c,e,g**) and CSP (**b,d,f,h**) plots are shown for SurA-WT bound to (**a,b**) peptide QMN (β2) (QMNKMGGFNLKYRYE), (**c,d**) peptide QMN_{ARY} (QMNKMGGFNLKARYE), (**e,f**) peptide QMN_{YRA} (QMNKMGGFNLKYRAE) and (**g,h**) peptide QMN_{ARA} (QMNKMGGFNLKARAE) (residues changed to Ala in red). Peaks which were broadened beyond detection in peptide-containing samples are indicated with an asterisk. Samples contained 5 μM SurA ± 200 μM peptide, 5 mM EDTA, 20 mM Tris-HCl, pH 8, at 25 ˚C. Signal to noise ratios were used for the calculation of errors in peak intensities in (a), (c), (e), and (g) (Methods). Peaks which are broadened below the noise are indicated by an asterisk. Source data are provided as a Source Data file.

	Repeat						Mean (± SD)
	1	2	3	4	5	6	
**OmpX N Labelled Alexa Fluor 488**	0.120	0.118	0.122	0.119	0.120	0.125	0.121 ± 0.002
**OmpX N Labelled ATTO 565**	0.082	0.066	0.087	0.100	0.088	0.108	0.080 ± 0.001
**OmpX C Labelled Alexa Fluor 488**	0.078	0.080	0.080	0.079	0.080	0.081	0.088 ± 0.015
**OmpX C Labelled ATTO 565**	0.076	0.082	0.076	0.086	0.084	0.085	0.081 ± 0.004

Supplementary Table 1. Steady state fluorescence anisotropy measurements. Single Cys variants of OmpX were labelled with either Alexa Fluor 488 or ATTO 565 on either the N- or C-termini and the fluorescence anisotropy measured (Supplementary Methods). The steady-state anisotropies of all the samples are similar and low, indicating good dye mobility, allowing changes in FRET efficiency to be attributed to changes in OmpX end-to-end distances.

**Protein**	**β-strand**	**Abbreviation**	**Residue numbers**	**Peptide**
OmpX	β2	QMN	40-54	QMNKMGGFNLKYRYE
OmpX	β3	GVI	61-75	GVIGSFTYTEKSRTA
OmpX	β4	NKN	81-95	NKNQYYGITAGPAYR
OmpX	β6	KHD	122-136	KHDTSDYGFSYGAGL
OmpX	β7	ENV	142-156	ENVALDFSYEQSRIR
OmpX	β8	SVD	157-172	SVDVGTWIAGVGYRF
Tmb2.3	β3	MVK	46-61	MVKAGYGWNNSSLNR
N/A	N/A	WEY	N/A	WEYIPNV
OmpX + mutation	β2	QMN-ARY	40-54	QMNKMGGFNLKARYE
OmpX + mutation	β2	QMN-YRA	40-54	QMNKMGGFNLKYRAE
OmpX + mutation	β2	QMN-ARA	40-54	QMNKMGGFNLKARAE
OmpX + mutation	β2	QMN-N-term	40-47	QMNKMGGF
OmpX + mutation	β3	QMN-C-term	48-54	NLKYRYE

Supplementary Table 2. Details of peptides used in this study. For the OmpX variants the residues changed are highlighted in red. The QMN N-term and C-term peptides are truncated at the C/N-termini, as indicated.

Core domain N-terminal mutants	P1 mutants	P2 mutants	Core domain C-terminal mutants
M46V	I179V	I280V	M400A
I70V	I181L	I290L	M402R
M71L	I202L	I297V	M414L
I75V	I217V	M298L	I425V
M76L	M231L	I310L	
I79L	I236L	I314L	
I80V	I242L	I348C	
M83L	I255V	M365L	
M87S	I259L	I378V	
I91V	I267L		
I100L			
I103V			
M109L			
M114L			
I132V			
M136L			
I137L			
I138L			
I150L			
I152V			

Supplementary Table 3. SurA isoleucine and methionine point mutants used in NMR peak assignment experiments. In each case the residue of interest was mutated to the most common substitution determined from a set of three domain SurA homologues curated from the PFAM database[9] and analysed using the ConsSurf server[10] (except for the I348C mutant).

Protein	Extinction coefficient at 280 nm (M^{-1} cm^{-1})
SurA-WT	30,940
SurA-ΔP1	25,440
SurA-ΔP1	19,940
SurA-core	14,440
OmpX	34,840
tOmpA	46,870
OmpF	54,210
tBamA	101,315

Supplementary Table 4. Extinction coefficients at 280 nm used for determining protein concentration.

Reagent	1 L	500 mL	250 mL
Na_2HPO_4 (anhydrous) (g)	13	6.5	3.25
KH_2PO_4 (g)	6	3	1.5
NaCl (g)	1	0.5	0.25
$^{15}NH_4Cl$ (g)	1	0.5	0.25
D-glucose (g)	2	1	0.5
Celtone Complete Medium (D) (mL)	10	5	2.5
$MgSO_4$ (anhydrous) (mg)	120	60	30
$CaCl_2$ (mg)	11	5.5	2.75
Biotin (mg)	10	5	2.5
Thiamine (mg)	10	5	2.5

Supplementary Table 5. Reagents used in NMR media for expression of deuterated, methyl-labelled SurA.

Reagent	Code
D_2O	DLM-4-99-1000
D-glucose (1,2,3,4,5,6,6-D7, 98%)	DLM-2062-1
Celtone Complete Medium (D)	CGM-1040-D
α-ketobutyric acid (Ile labelling)	CDLM-7318
L-methionine (Met labelling)	CDLM-8885

Supplementary Table 6. Cambridge Isotopes Ltd (CIL) codes for reagents used in expression of deuterated, methyl-labelled SurA (Ile and Met residues).

Supplementary Methods

smFRET Instrumentation

smFRET experiments were performed on a custom-built confocal instrument using a Nikon Eclipse TE300 inverted microscope. Single molecules which diffused through the confocal volume were excited using PIE (pulsed interleaved excitation) with a 480 nm (PiL048XSM, Advanced Laser Diode Systems) and a 561 nm (PDL 800-D, PicoQuant) laser producing a total excitation frequency of 40 MHz[11]. The two laser beams were combined with a dichroic mirror (Di02-R561-25x36, Semrock) before being coupled into a single mode fibre (P3-460B-FC-1, Thorlabs Inc.) using an achromatic collimator (PAF2A-A15A, Thorlabs Inc.). The laser from the fibre was collimated (60FC-4-A11-01, Schäfter+Kirchhoff GmbH) and reflected from a dichroic mirror (Di03-R488/561-t1-25x36, Semrock) into a 60x 1.2 numerical aperture water immersion objective (UPLSAPO60XW, Olympus). Light emitted from the sample was recollected by the same objective before being focused by the tube lens through a 100 μm pinhole (P100D Thorlabs Inc.) and collimated (AC254-050-A, Thorlabs Inc.). The emission light is then split into two using a dichroic mirror (Di03-R561-t3-25x36, Semrock) before band-pass filters remove further excitation light in each channel (ET525/50m & ET605/52m, Chroma Technology GmbH). The two emission pathways are then focused by a lens (AC254-050-A, Thorlabs Inc.) onto two single-photon avalanche diodes (PD-100-CTD & PD-050-CTD, PicoQuant). Signal from the detectors and a sync signal from the laser was sent to a time-correlated single-photon counting data acquisition card (TimeHarp 260 PICO, PicoQuant). SymPhoTime64 software (PicoQuant) was used to acquire data.

Fluorescence Anisotropy

Steady state fluorescence anisotropy was measured using a PTI QuantaMaster (Horiba Scientific). Prior to measurement samples were passed through a 30 kDa molecular weight cut off centrifugal filter to remove aggregates (Vivaspin 500, Sartorius). Samples were measured at 21 °C in a quartz glass cuvette (109004F-10-40, Hellma) at a volume of 1 ml and concentration of ~1 nM. The sample buffer was the same used for single-molecule FRET experiments without the antifading agent Trolox (20 mM Tris-HCl, 0.1 M urea, 0.02% (v/v) Tween-20, pH 8.0). For OmpX labelled with Alexa Fluor 488 the samples were excited at 495 nm and emission collected at 519 nm. For OmpX labelled with ATTO 565 the samples were excited at 564 nm and emission collected at 590 nm. The excitation and emission slits for all samples were set to 8 nm and 15 nm respectively. The grating correction factor was measured for each sample before the anisotropy measurement. Data acquisition and analysis were both performed using PTI FelixGX (v4.9.0.10243, Horiba Scientific).

Supplementary References

1. Bitto, E. & McKay, D.B. Crystallographic structure of SurA, a molecular chaperone that facilitates folding of outer membrane porins. *Structure* **10**, 1489-98 (2002).
2. Vogt, J. & Schulz, G.E. The structure of the outer membrane protein OmpX from Escherichia coli reveals possible mechanisms of virulence. *Structure* **7**, 1301-9 (1999).
3. Arora, A., Abildgaard, F., Bushweller, J.H. & Tamm, L.K. Structure of outer membrane protein A transmembrane domain by NMR spectroscopy. *Nat Struct Biol* **8**, 334-338 (2001).
4. Yamashita, E., Zhalnina, M.V., Zakharov, S.D., Sharma, O. & Cramer, W.A. Crystal structures of the OmpF porin: function in a colicin translocon. *EMBO J* **27**, 2171-80 (2008).
5. Ni, D. et al. Structural and functional analysis of the β-barrel domain of BamA from Escherichia coli. *The FASEB Journal* **28**, 2677-2685 (2014).
6. Bitto, E. & McKay, D.B. The periplasmic molecular chaperone protein SurA binds a peptide motif that is characteristic of integral outer membrane proteins. *J Biol Chem* **278**, 49316-22 (2003).
7. Xu, X., Wang, S., Hu, Y.X. & McKay, D.B. The periplasmic bacterial molecular chaperone SurA adapts its structure to bind peptides in different conformations to assert a sequence preference for aromatic residues. *J Mol Biol* **373**, 367-81 (2007).
8. Vorobieva, A.A. et al. De novo design of transmembrane β barrels. *Science* **371**, eabc8182 (2021).
9. Humes, J.R. et al. The role of SurA PPIase domains in preventing aggregation of the outer-membrane proteins tOmpA and OmpT. *J Mol Biol* **431**, 1267-1283 (2019).
10. Ashkenazy, H. et al. ConSurf 2016: an improved methodology to estimate and visualize evolutionary conservation in macromolecules. *Nucleic Acids Res* **44**, W344-W350 (2016).
11. Müller, B.K., Zaychikov, E., Bräuchle, C. & Lamb, D.C. Pulsed interleaved excitation. *Biophys J* **89**, 3508-22 (2005).
