# Supplementary material for: Dual client binding sites in the ATP-independent chaperone SurA
Source: Nat Commun. 2024 Sep 14;15:8071. doi: 10.1038/s41467-024-52021-1 (PMC11401910; doi:10.1038/s41467-024-52021-1)
Supplement: Supplementary file 3 — Reporting Summary [file 41467_2024_52021_MOESM3_ESM.pdf]

nature portfolio

Corresponding author(s): *Anastasia Zhuravleva, Sheena E. Radford*

Last updated by author(s): *Aug 1, 2024*

Reporting Summary

Nature Portfolio wishes to improve the reproducibility of the work that we publish. This form provides structure for consistency and transparency in reporting. For further information on Nature Portfolio policies, see our Editorial Policies and the Editorial Policy Checklist.

Statistics

For all statistical analyses, confirm that the following items are present in the figure legend, table legend, main text, or Methods section.

n/a	Confirmed	
☐	☒	The exact sample size (*n*) for each experimental group/condition, given as a discrete number and unit of measurement
☐	☒	A statement on whether measurements were taken from distinct samples or whether the same sample was measured repeatedly
☒	☐	The statistical test(s) used AND whether they are one- or two-sided<br>*Only common tests should be described solely by name; describe more complex techniques in the Methods section.*
☒	☐	A description of all covariates tested
☒	☐	A description of any assumptions or corrections, such as tests of normality and adjustment for multiple comparisons
☐	☒	A full description of the statistical parameters including central tendency (e.g. means) or other basic estimates (e.g. regression coefficient) AND variation (e.g. standard deviation) or associated estimates of uncertainty (e.g. confidence intervals)
☐	☒	For null hypothesis testing, the test statistic (e.g. *F*, *t*, *r*) with confidence intervals, effect sizes, degrees of freedom and *P* value noted<br>*Give P values as exact values whenever suitable.*
☒	☐	For Bayesian analysis, information on the choice of priors and Markov chain Monte Carlo settings
☐	☒	For hierarchical and complex designs, identification of the appropriate level for tests and full reporting of outcomes
☒	☐	Estimates of effect sizes (e.g. Cohen's *d*, Pearson's *r*), indicating how they were calculated

Our web collection on statistics for biologists contains articles on many of the points above.

Software and code

Policy information about availability of computer code

Data collection	*NMR data was collected using Bruker TopSpin v3.5 with use of the nmrlib plugin. FRET data was acquired using the SymPhoTime64 software v2.8.5759 and data was converted into HDF5 files using phconvert v0.9.*
Data analysis	*NMR data was processed with NMRPipe and analysed with CcpNmr Analysis v2.5.1 and ploting was carried out using in-house Python 3 scripts using the SciPy, v1.5.0, Matplotlib v3.2.2, NumPy v1.18.5, Nmrglue v0.8, and Biopython v1.79. FRET data was analysed using FRETBursts v0.7.1*

For manuscripts utilizing custom algorithms or software that are central to the research but not yet described in published literature, software must be made available to editors and reviewers. We strongly encourage code deposition in a community repository (e.g. GitHub). See the Nature Portfolio guidelines for submitting code & software for further information.

Data

Policy information about availability of data

All manuscripts must include a data availability statement. This statement should provide the following information, where applicable:

- Accession codes, unique identifiers, or web links for publicly available datasets
- A description of any restrictions on data availability
- For clinical datasets or third party data, please ensure that the statement adheres to our policy

Assignments for Ile and Met peaks in the 2D 1H-13C methyl-TROSY NMR spectrum of SurA-WT have been deposited in the Biological Magnetic Resonance Bank

(BMRB Entry ID 52180) (https://doi.org/10.13018/BMR52180). Raw smFRET data are available at the University of Leeds data repository (https://doi.org/10.5518/1423). The source data underlying Figs. 1-5, and Supplementary Figs. 1-3, 5-11, and 13-17 are provided in the Source Data file. All other data are available from the corresponding author on request.

Research involving human participants, their data, or biological material

Policy information about studies with human participants or human data. See also policy information about sex, gender (identity/presentation), and sexual orientation and race, ethnicity and racism.

Reporting on sex and gender	n/a
Reporting on race, ethnicity, or other socially relevant groupings	n/a
Population characteristics	n/a
Recruitment	n/a
Ethics oversight	n/a

Note that full information on the approval of the study protocol must also be provided in the manuscript.

Field-specific reporting

Please select the one below that is the best fit for your research. If you are not sure, read the appropriate sections before making your selection.

[x] Life sciences   [ ] Behavioural & social sciences   [ ] Ecological, evolutionary & environmental sciences

For a reference copy of the document with all sections, see nature.com/documents/nr-reporting-summary-flat.pdf

Life sciences study design

All studies must disclose on these points even when the disclosure is negative.

Sample size	*No statistical methods were used to predetermine sample size. Sample sizes were chosen in agreement with established procedures in the field.*
Data exclusions	*No data was excluded from the analysis*
Replication	*smFRET experiments and biochemical assays were replicated (n > 2, as described in the Methods), and NMR experiments were repeated at least twice to ensure reproducibility of the findings. The number of independent replicates for each experiment are specified in the respective Figure legends All attempts at replication were successful.*
Randomization	*The experiments were not randomized, in agreement with established procedures in the field.*
Blinding	*The investigators were not blinded to allocation during experiments and outcome assessment, in agreement with established procedures in the field.*

Reporting for specific materials, systems and methods

We require information from authors about some types of materials, experimental systems and methods used in many studies. Here, indicate whether each material, system or method listed is relevant to your study. If you are not sure if a list item applies to your research, read the appropriate section before selecting a response.

Materials & experimental systems

n/a	Involved in the study	
[x]	[ ]	Antibodies
[x]	[ ]	Eukaryotic cell lines
[x]	[ ]	Palaeontology and archaeology
[x]	[ ]	Animals and other organisms
[x]	[ ]	Clinical data
[x]	[ ]	Dual use research of concern
[x]	[ ]	Plants

Methods

n/a	Involved in the study	
[x]	[ ]	ChIP-seq
[x]	[ ]	Flow cytometry
[x]	[ ]	MRI-based neuroimaging

Plants

Seed stocks	*Report on the source of all seed stocks or other plant material used. If applicable, state the seed stock centre and catalogue number. If plant specimens were collected from the field, describe the collection location, date and sampling procedures.*
Novel plant genotypes	*Describe the methods by which all novel plant genotypes were produced. This includes those generated by transgenic approaches, gene editing, chemical/radiation-based mutagenesis and hybridization. For transgenic lines, describe the transformation method, the number of independent lines analyzed and the generation upon which experiments were performed. For gene-edited lines, describe the editor used, the endogenous sequence targeted for editing, the targeting guide RNA sequence (if applicable) and how the editor was applied.*
Authentication	*Describe any authentication procedures for each seed stock used or novel genotype generated. Describe any experiments used to assess the effect of a mutation and, where applicable, how potential secondary effects (e.g. second site T-DNA insertions, mosiacism, off-target gene editing) were examined.*
